# Transcriptomic Analysis Reveals That Municipal Wastewater Effluent Enhances *Vibrio vulnificus* Growth and Virulence Potential

**DOI:** 10.3389/fmicb.2021.754683

**Published:** 2021-10-25

**Authors:** Karlen Enid Correa Velez, Robert Sean Norman

**Affiliations:** ^1^Department of Environmental Health Sciences, University of South Carolina, Columbia, SC, United States; ^2^Department of Environmental Health Sciences, NIEHS Center for Oceans and Human Health and Climate Change Interactions, University of South Carolina, Columbia, SC, United States

**Keywords:** *Vibrio vulnificus*, wastewater, transcriptomic, climate change, stress response

## Abstract

*Vibrio vulnificus* is an opportunistic pathogen indigenous to estuarine and marine environments and associated with aquatic organisms. *Vibrio vulnificus* is of utmost importance because it causes 95% of the seafood-related deaths in the United States due to rapid progression of septicemia. Changes in environmental parameters associated with climate change and coastal population expansion are altering geographical constraints, resulting in increased *Vibrio* spread, exposure, and rates of infection. In addition, coastal population expansion is resulting in increased input of treated municipal sewage into areas that are also experiencing increased *Vibrio* proliferation. This study aimed to better understand the influence of treated sewage effluent on effluent-receiving microbial communities using *Vibrio* as a model of an opportunistic pathogen. Integrated transcriptomic approaches were used to analyze the changes in overall gene expression of *V. vulnificus* NBRC 15645 exposed to wastewater treatment plant (WWTP) effluent for a period of 6h using a modified seawater yeast extract media that contained 0, 50, and 100% filtered WWTP effluent. RNA-seq reads were mapped, annotated, and analyzed to identify differentially expressed genes using the Pathosystems Resource Integration Center analysis tool. The study revealed that *V. vulnificus* responds to wastewater effluent exposure by activating cyclic-di-GMP-influenced biofilm development. Also, genes involved in crucial functions, such as nitrogen metabolism and bacterial attachment, were upregulated depending on the presence of treated municipal sewage. This altered gene expression increased *V. vulnificus* growth and proliferation and enhanced genes and pathways involved in bacterial survival during the early stages of infection in a host. These factors represent a potential public health risk due to exposure to environmental reservoirs of potentially *Vibrio* strains with enhanced virulence profiles in coastal areas.

## Introduction

Climate change, sea warming, and increasing ocean pollution have been recognized in the Human Health and Ocean Review (Landrigan et al., 2020) as contributing factors to increased frequency of *Vibrio*-illness and its spread into previously unaffected areas. *Vibriosis*, an infection caused by *Vibrio* species, can present multiple clinical manifestations including gastroenteritis and wound infections that can develop further into acute septicemia in immunocompromised individuals. This opportunistic pathogen grows preferentially in warm aquatic environments (>18°C) with salinity ranging from approximately 5 to 25ng/L NaCl. These narrow growth parameters have historically acted as environmental constraints to the spread of *Vibrio* in natural ecosystems. However, changes in environmental conditions associated with climate change, such as temperature and sea level rise, are altering geographical constraints and increasing *Vibrio* spread and rates of infection. Multiple reports (Newton et al., 2012; Baker-Austin et al., 2013; Vezzulli et al., 2013) have documented that *Vibrio* infections are increasing worldwide. In the United States, the most recent report from the Foodborne Diseases Active Surveillance Network (Tack et al., 2019) indicated that the 2018 incidence of *Vibrio* infections increased 109% and the number of diagnosed infections by 311% as compared with 2015–2017 incidence. One of the most common non-cholera *Vibrio* species that represent a risk to human health is *Vibrio vulnificus*. *Vibrio vulnificus* is a Gram-negative bacterium indigenous to estuarine, and coastal environments that can be found in seawater, sediments, and seafood products (Oliver et al., 2013; Baker-Austin et al., 2017). This bacterium is an invasive pathogen for humans and aquatic animals and in both can be transmitted by contact or ingestion. *Vibrio vulnificus* strains that have been shown to cause infection in humans possess virulence factors, such as acid neutralization, capsular polysaccharide expression, iron acquisition, cytotoxicity, motility, and expression of proteins involved in attachment and adhesion that have been associated with increased pathogenicity (Jones and Oliver, 2009). *Vibrio vulnificus* is a diversified species that have been classified in five phylogenetic lineages and one pathovar based on the single-nucleotide polymorphisms present in the core genome (Roig et al., 2018). This species comprises avirulent and virulent strains and that have been classified into three biotypes (Oliver, 2015). Transcriptomic studies analyzing *V. vulnificus* strains from different groups of biotypes have confirmed that diverse external signals, such as environmental cues, nutrient level, and temperature, may trigger a host-adapted virulence profile (Bisharat et al., 2013; Williams et al., 2014; Pajuelo et al., 2016; Hernández-Cabanyero et al., 2019, 2020). The differentially expressed genes in these studies included genes involved in flagellar components, GGDEF family protein, iron acquisition system, bacterial toxins (RtxA1 and VvhA), and others.

In addition to the climate-linked changes that are increasing *Vibrio* proliferation and spread, the population of people inhabiting areas of increased potential of *Vibrio* exposure is also increasing. The National Oceanic and Atmospheric Administration and U.S. Census Bureau (2013) documented that the coastal population in the United States has increased exponentially in recent decades; in 2010, approximately 123.3 million people resided in coastal shoreline counties. Population density is significantly higher in coastal regions than in-land regions worldwide, and this coastal migration trend is likely to continue (Small and Nicholls, 2003; Balk et al., 2009; Neumann et al., 2015). Population growth and development in these regions generate high pressure on coastal ecosystems due to anthropogenic pollution (Crossland et al., 2005; Patterson and Hardy, 2008). Furthermore, growth within this urban setting increases the necessity of additional sewage collection infrastructure with wastewater treatment plants (WWTPs) providing end-point treatment before environmental discharge of treated wastewater effluent. For instance, in 2012, there were approximately 14,748 publicly owned wastewater treatment plants in the United States serving 238.2 million individuals (76% of the population; United States Environmental Protection Agency, 2016). Additionally, the number of secondary treatment plants is projected to increase by 4% by 2023, resulting in increased environmental discharges. While, WWTPs significantly improve water quality, they have also been shown to act as reservoirs of antibiotic resistance with antibiotics, antibiotic-resistant bacteria (ARB), and antibiotic resistance genes (ARGs) observed in final treated effluent (Rizzo et al., 2013; Xu et al., 2015; Guo et al., 2017). It has been shown that ARGs from WWTPs are subsequently transferred into surrounding ecosystems through the release of treated effluent (Chu et al., 2018). Also, studies have demonstrated that WWTP effluent can alter and shift the microbial community composition of the surrounding ecosystems due to the introduction of non-native bacteria. The organisms associated with WWTP processes, nutrient cycling, and fecal indicators are often identified downstream of effluent sources and mixed with the autochthonous communities of the effluent-receiving ecosystems (Tang et al., 2016; Price et al., 2018; Mansfeldt et al., 2020; Pascual-Benito et al., 2020).

Given that increases in ocean temperature and sea level coupled with increases in human activity in coastal waters support greater Vibrio-human interactions (Froelich and Daines, 2020), it is important to examine mechanisms within this socio-ecological coupled system that may further strengthen this interaction. WWTP discharges into areas capable of supporting *Vibrio* proliferation is one potential mechanism that may alter *Vibrio* growth patterns and virulence profiles. This study aimed to better understand this potential mechanism by exposing a well-characterized strain of *V. vulnificus* to different concentrations of treated wastewater effluent and using transcriptomic analysis to identify genes that are differentially regulated in the presence of treated wastewater. A better understanding of how *V. vulnificus* responds to wastewater discharges will allow for the construction of more detailed models to predict potential *Vibrio* outbreaks and will increase health risk awareness in coastal regions.

## Materials and Methods

### Strains and Growth Conditions

The type strain *V. vulnificus* NBRC 15645 (ATCC 27562) was used as the bacterial model for this study and was initially isolated from a blood sample of an individual infected after exposure to seawater contaminated with lactose positive, halophilic vibrios (Reichelt et al., 1976). For the wastewater exposure experiments, 4L of final treated wastewater effluent were collected from the Columbia Metropolitan Wastewater Treatment Plant located in South Carolina, United States. A full list of the chemical analysis of the wastewater effluent used in this study, including the environmental parameters and potential contaminants, can be found in the Supplementary Table S1. Effluent was filtered through a 0.22μm polyethersulfone (PES) membrane and stored in the dark at 4°C until used to establish effluent-amended growth media. To examine the effect of wastewater effluent on bacterial growth, *V. vulnificus* was grown at 25°C in 96 well plates containing four replicates of 150μl of Modified Seawater Yeast Extract (MSYE; Oliver and Colwell, 1973) with 0% effluent (MSYE reconstituted with 100% DI water and no effluent), 50% effluent (MSYE reconstituted with 50% effluent and 50% DI water), and 100% effluent (MSYE reconstituted with 100% effluent and 0% DI water). Modified Seawater Yeast Extract was used to provide sufficient carbon in order to assess the effects of other factors on bacterial growth. The optical density at 600nm (OD_600_) of each replicate was measured hourly to determine bacterial growth over 24h using a Victor X3 plate reader (PerkinElmer, Waltham, MA, United States). Gaussian process regression was performed on background subtracted OD data to model growth curves and model-predicted ODs were used to estimate growth parameters for each treatment using AMiGA (Midani et al., 2021). The mean squared error (MSE) of model fit to the growth curves for the 0, 50, and 100% effluent conditions was 0.008, 0.006, and 0.004, respectively.

### Wastewater Exposure Transcriptomic Study

*Vibrio vulnificus* was initially grown in MSYE at 25°C until it reached the exponential phase (OD 0.4). Around 1ml of the *V. vulnificus* culture was then used as inoculum for the different wastewater effluent exposure conditions in a ratio of 1:100 (v/v; Supplementary Figure S1). Exposure conditions consisted of MSYE with 0, 50, and 100% effluent. Briefly, filtered effluent or DI water was used to reconstitute the culture media as described above, allowing a constant level of the media components across all experimental conditions. Exposure cultures (100ml) were grown in 250ml flasks for 24h at 25°C with shaking at 220rpm, and three replicates were performed for each condition. At 6, 12, and 24h of growth, the OD_600_ of the cultures was measured using a Victor X3 plate reader to determine bacterial growth stage and 5ml of each culture was collected, centrifuged at 5,000 *g* for 10min, and washed twice with MSYE. After washing, the cells were resuspended in 2ml of MSYE and used for nucleic acid and protein extraction.

### RNA Extraction, Library Preparation, and Sequencing

DNA, RNA, and protein were extracted using the Allprep Bacterial DNA/RNA/Protein kit (Qiagen, Germantown, MD, United States) following the manufacturer’s instructions. Briefly, three replicates of each condition were performed, and total DNA, RNA, and proteins were extracted from 2ml cell resuspension of 6, 12, and 24h culture growth. Gene expression analysis was limited to the 6h time point (exponential phase; OD_600_: 0.214 at 0%, 0.221 at 50%, and 0.231 at 100% effluent) to minimize the effect of growth stage-dependent gene expression. DNA and protein were preserved for future analysis. RNA quantity was measured using a NanoDrop spectrophotometer (Thermo Scientific, Waltham, MA, United States) and RNA quality was assessed using an Agilent Bioanalyzer (Agilent Technologies, Santa Clara, CA, United States) using the RNA High Sensitivity Assay. Ribosomal RNA was depleted from the total RNA using the NEBNext rRNA Depletion Kit (New England Biolabs, Ipswich, MA, United States) and the quality and quantity of the remaining mRNA were assessed using an Agilent Bioanalyzer. Purified mRNA was used to prepare individually barcoded RNA-seq libraries using the NEBNext Ultra II Directional RNA Library Prep Kit (New England Biolabs, Ipswich, MA, United States) according to the manufacturer’s instructions for intact RNA [RNA Integrity Number (RIN)>7]. All the samples used in this study had a RIN≥9.7. The RNA-seq libraries were sequenced on an Illumina HiSeq DNA sequencer with 2×250bp paired end reads. All raw sequencing data were deposited into the GenBank Sequence Read Archive (SRA) database under the BioProject accession number *PRJNA747618*.

### RNA-Seq Analysis

Following sequencing, raw reads were quality and adapter trimmed using fastp (v.0.20.0) with the following parameters: *-3* (drop the bases in the 3′ end if the mean quality is lower of the cut mean quality) and *-y* (low complexity filter; Chen et al., 2018). Cleaned RNA-seq reads were aligned against the *V. vulnificus* NBRC 15645 reference genome (GenBank Accessions: CP012882.1 and CP012881.1), annotated, and analyzed to find differentially expressed genes using the Tuxedo strategy in the Pathosystems Resource Integration Center (PATRIC) analysis tool (Wattam et al., 2017). The Tuxedo strategy used Bowtie 2, Cufflinks, and Cuffdiff to align, assemble, and compare samples, respectively (Trapnell et al., 2012). The default parameters were used for the transcript analysis. Cuffdiff calculated the log_2_ fold change in Fragments Per Kilobase of transcript per Million mapped reads (FPKM) and then the fold change significance. The 0% effluent condition (control) was treated as the baseline condition for the analysis. The statistical significance threshold for differential gene expression was an FDR-adjusted *p*-value less than or equal to 0.05.

To visualize differences among the treatments in an overall analysis, a principal component analysis (PCA) was generated using the “prcomp” function and visualized using the “ggbiplot’ package (Vu, 2011) of the R software (R Core Team, 2021). The PCA values were calculated from the gene expression (FPKM values) across samples conditions.

## Results

### Wastewater Effluent Alters *V. vulnificus* Growth Patterns

It was observed that bacterial growth was enhanced when grown in media containing 50 and 100% effluent as compared to media with 0% effluent (Supplementary Figure S2). The growth rate of *V. vulnificus* increased from 0.20 in the 0% effluent condition to 0.24 and 0.26 in the 50 and 100% effluent conditions, respectively (Table 1). Additionally, bacterial cultures grown in both 50 and 100% effluent conditions showed significant reductions in doubling time (2.9 and 2.7h, respectively) as compared to the 0% effluent condition (3.5h). The increased growth rate and decreased doubling time resulted in a significant decrease in the time delay (Lag Time) needed to enter exponential phase from 4.8h for 0% effluent to 2.8h and 2.2h for 50 and 100% effluent conditions, respectively. Overall, the increased area under the curve (AUC) for the 50 and 100% effluent conditions (25.6 and 26.5, respectively) as compared to 0% effluent (22.1) suggests that the wastewater effluent conditions support enhanced *V. vulnificus* growth.

**Table 1:** Growth rate of *Vibrio vulnificus* in 0, 50, and 100% wastewater treatment plant (WWTP) effluent condition.

Growth parameter^*^	Modified seawater yeast extract condition			Significance^**^
	0% effluent	50% effluent	100% effluent	
Area under curve (log)+CI	21.119 [21.720–22.518]	25.582 [25.186–25.977]	26.491 [26.181–26.802]	a,b,c
Lag time+CI	4.818 [4.535–5.102]	2.834 [2.574–3.094]	2.225 [2.004–2.447]	a,b,c
Growth Rate+CI	0.2 [0.184–0.216]	0.242 [0.221–0.262]	0.258 [0.233–0.283]	a,b
Doubling Time+CI	3.472 [3.190–3.754]	2.874 [2.634–3.115]	2.691 [2.428–2.955]	a,b

* AUC shows overall growth.

Lag Time: time delay needed to enter exponential growth; CI, confidence interval.

** a=0% vs. 50% effluent; b=0% vs. 100% effluent; and c=50% vs. 100% effluent.

### Wastewater Effluent Alters *V. vulnificus* Gene Expression

RNA-seq was performed to examine how wastewater effluent changes the gene expression profile of *V. vulnificus* and to identify the genes involved in the bacterial exposure response. To minimize the effect of growth stage-dependent gene expression across exposure conditions, transcriptomic analysis was limited to the 6h time point, the point at which all cultures were in the exponential phase of growth. Total RNA isolated from three biological replicates of *V. vulnificus* exposed to each condition was sequenced on an Illumina HiSeq 2000, resulting in approximately 10.9–19.2 million total sequence reads per replicate (Supplementary Table S2). Following DNA sequence quality filtering and trimming, approximately 10.7–18.9 million reads were retained and used in subsequent RNA-seq analysis. The *V. vulnificus* NBRC 15645 reference genome was annotated using the PATRIC analysis tool (Wattam et al., 2017) and 4,564 open reading frames (ORFs) were identified. The cleaned sequences from each exposure condition were then mapped against the annotated reference genome and transcript abundances compared. As compared against the control (0% effluent), growth in 50% effluent resulted in significant changes in the expression of 742 genes 412 upregulated and 330 downregulated (FDR-adjusted value of *p*≤0.05; Fold Change Log_2_≥|1|; Table 2). The log_2_ fold change in FPKM for the 50% effluent exposure varied between 1.00 and 8.03 for upregulated genes and from −1.00 to −5.26 for downregulated genes. Similarly, growth in 100% effluent resulted in 487 upregulated genes and 528 downregulated genes as compared to the 0% effluent control. The log_2_ fold change in FPKM for the 100% effluent exposure varied between 1.00 and 7.46 for upregulated (FDR-adjusted value of *p*≤0.05; Fold Change Log_2_≥|1|) and from −1.00 to −5.52 for downregulated genes (FDR-adjusted value of *p*≤0.05; Fold Change Log_2_≥|1|). A complete list of significant differentially expressed genes in *V. vulnificus* when grown in 50 and 100% treated municipal wastewater can be found in Supplementary Table S3.

**Table 2:** *Vibrio vulnificus* differentially expressed genes in wastewater by functional categories.

Category	50% WWTP effluent		100% WWTP effluent	
	Upregulated^*^	Downregulated^*^	Upregulated^*^	Downregulated^*^
Cell envelope	6 (4)	13 (3)	8 (6)	27 (10)
Cellular processes	44 (16)	54 (8)	57 (23)	56 (14)
DNA processing	5 (2)	31 (5)	8 (2)	41 (12)
Energy	64 (34)	69 (32)	57 (38)	89 (54)
Membrane transport	84 (51)	56 (29)	103 (53)	68 (35)
Metabolism	201 (91)	213 (75)	160 (92)	235 (107)
Miscellaneous	2 (2)	1 (0)	2 (1)	2 (1)
Protein processing	20 (5)	108 (35)	20 (3)	134 (71)
Regulation and cell signaling	18 (16)	11 (0)	18 (11)	11 (3)
RNA processing	39 (12)	59 (11)	47 (18)	61 (21)
Stress response, defense and virulence	72 (27)	117 (30)	112 (44)	129 (52)
Uncategorized	438 (152)	355 (102)	513 (196)	410 (148)
Total	993 (412)	1,087 (330)	993 (412)	1,087 (330)

* FDR-adjusted value of *p*≤0.05.

The number in parenthesis (#) represents the differentially expressed genes with Fold Change Log_2_≥|1|.

A PCA was performed to compare the expression profiles between conditions of 0, 50, and 100% effluent. The PCA results indicated that the global expression profile differs for each experimental condition and shows similarity among the global expression profile obtained for each biological replicate (Supplementary Figure S3). The first component (PC1) explained 40.3% of the variation, and the second component (PC2) explained 27.2%.

**Figure 1:**
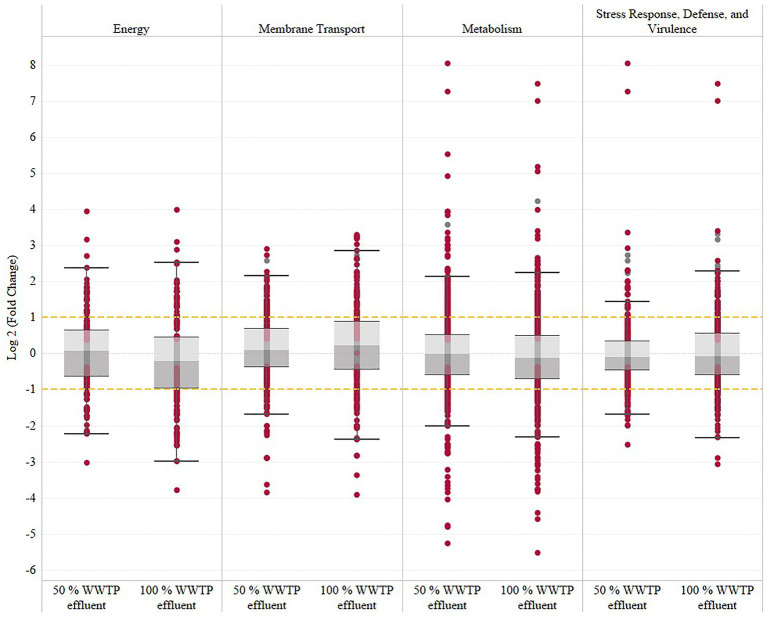
Summary of *V. vulnificus* transcriptomic response to wastewater effluent exposure across the energy, membrane transport, metabolism and stress response, defense, and virulence categories established by the PATRIC analysis tool. This table shows the overall log_2_ fold change at 50 and 100% wastewater effluent relative to the 0% wastewater effluent control. The significantly regulated genes (FDR-adjusted value of *p*≤0.05) are represented with garnet dots, and gray dots represent the non-significant genes.

The transcriptomic analysis revealed a consistent functional enrichment pattern in the genes related to metabolism, energy, and membrane transport functional categories established by the PATRIC analysis tool (Figure 1; Supplementary Figure S4). Also, genes involved in adherence were upregulated at 50 and 100% WWTP effluent, reflecting a constant cycle of attachment where the bacteria alternate between planktonic and biofilm modes of growth. The biofilm dynamic is further supported by the upregulation of the signaling pathway of the intracellular second messenger cyclic diguanylate [cyclic-di-GMP (3′,5′-cyclic diguanylic acid)], which levels are controlled by the upregulation of diguanylate cyclases (GGDEF domain) and phosphodiesterases (EAL domain; Supplementary Table S4).

### Metabolism

A total of 166 metabolism-related genes showed significant changes in expression within the 50% wastewater condition as compared to 0% wastewater: 91 genes were upregulated, and 75 genes were downregulated (Table 2; Figure 1). Similar trends were observed in the 100% wastewater condition with 199 genes showing significant differential regulation (92 upregulated and 107 downregulated). The most highly upregulated metabolism-related genes upon exposure to 50 and 100% wastewater effluent were genes involved in nitrogen metabolism (Supplementary Figure S5). The upregulated genes associated with nitrate/nitrite metabolism included genes that encode periplasmic nitrate reductase (fig|1219061.36.peg.3842), cytochrome C_552_ nitrite reductase subunit (fig|1219061.36.peg.292), and glutamine synthetase type I (fig|1219061.36.peg.1250). In nitrogen assimilation, the nitrate reductase reduces nitrate to nitrite, and then nitrite reductase transforms nitrite into ammonia. The glutamine synthetase catalyzes the reaction of glutamate and ammonia to form glutamine. Subsequently, glutamine is metabolized to aminosugars. Furthermore, six upregulated genes related to nitrogen metabolism are potentially involved in nitrosative stress defense and were upregulated in wastewater exposure conditions: nitric oxide (NO) dioxygenase, NrfA (cytochrome C_552_ nitrite reductase subunit), NrfD (cytochrome C nitrite reductase subunit), NnrS (putative heme−/copper-containing transmembrane protein), and NsrR (nitrite-sensitive transcription repressor). NO dioxygenase is induced in nitrosative stress, and it detoxifies high levels of NO under anaerobic conditions by oxidizing it to the less toxic form, NO_3_^−^. Also, in response to NO exposure, NsrR acts as an NO-response transcription regulator that relieves the repression of NO dioxygenase.

Decreased expression of genes involved in phosphate metabolism was observed in both wastewater effluent conditions. Most of the downregulated genes were related to the inorganic phosphate (P_i_)-specific transport (Pst) system: *pstS*, *pstA*, *pstC*, and *pstB*. The Pst system is a periplasmic protein-dependent transporter expressed under P*i* starvation and serves as a scavenger of P*i* residues. This downregulation supports increased availability of phosphate due to the addition of wastewater. Other genes involved in phosphate metabolism included the alkaline phosphatase and the Pho regulon (*phoU*, *phoB*, and *phoR*), which were downregulated or slightly upregulated (Fold Change Log_2_≤2).

Additionally, the genes involved in the utilization of galactose were upregulated in all *V. vulnificus* samples exposed to wastewater effluent. The carbon starvation protein A and maltose metabolism genes were downregulated upon exposure to wastewater effluent. Some genes related to the metabolism of amino acids, such as histidine, arginine, cysteine, succinate, and glutamine, were upregulated upon exposure to wastewater effluent conditions. A similar trend was observed with the aldehyde dehydrogenase (fig|1219061.36.peg.3697) in both 50 and 100% wastewater effluent conditions. Genes involved in iron metabolism were not differentially regulated upon wastewater exposure.

### Energy

Energy metabolism in bacteria can be influenced by multiple factors, such as temperature, organic matter, nutrients, and metal ions. Heterotrophic bacteria, including *V. vulnificus*, obtain energy from the oxidation of organic compounds, such as carbohydrates, lipids, and proteins, resulting in the synthesis of adenosine triphosphate (ATP). *Vibrio vulnificus* exposed to wastewater effluent upregulated 34 genes (50% wastewater condition) and 38 genes (100% wastewater condition) involved in energy metabolism (Table 1; Supplementary Figure S6). Most of the upregulated genes were related to bacterial central metabolism, which is supported by the increased energy requirements (ATP/GTP) needed for enhanced growth of *Vibrio* in the presence of wastewater. The upregulated genes included acetyl-CoA synthetase (fig|1219061.36.peg.1540), succinate dehydrogenase iron-sulfur protein (fig|1219061.36.peg.538), aldehyde dehydrogenase (fig|1219061.36.peg.3697), and C-type cytochromes. The most upregulated gene in both 50 and 100% conditions was aldehyde dehydrogenase. Aldehyde dehydrogenase is involved in multiple metabolic pathways, such as histidine and alanine metabolism and fatty acid degradation. The acetyl-CoA synthetase forms the acetyl-CoA used in the tricarboxylic acid cycle to produce ATP, and C-type cytochromes are crucial in the electron transport chain.

The availability of substrates, such as carbon and nitrogen sources, can regulate bacterial gene expression. The genes encoding uptake and metabolism of a substrate can be repressed if they are limited or absent in the surrounding environment. For example, the phosphoenolpyruvate:carbohydrate phosphotransferase system (PTS) catalyzes the uptake and phosphorylation of numerous carbohydrates and the availability of carbohydrates is reflected in PTS component expression (Kotrba et al., 2001; Deutscher et al., 2014). In the 50 and 100% wastewater conditions, the genes associated with fructose-, glucose-, ascorbate-, and mannose-specific PTS systems were downregulated as well as genes encoding glucose-6-phosphate isomerase (fig|1219061.36.peg.1694) and phosphoenolpyruvate-protein phosphotransferase (enzyme I) of PTS system (fig|1219061.36.peg.590). These changes further suggest that exposure to wastewater effluent causes an overall shift in *V. vulnificus* energy and metabolism.

### Membrane Transport

Within the membrane transport category, a significant fraction of the upregulated genes corresponded to solute binding-protein-dependent systems, particularly ATP-binding cassette (ABC) transporters and secondary Tripartite ATP-independent periplasmic (TRAP) transporters (Supplementary Figure S7). In Gram-negative bacteria, outer membrane proteins play a significant role in acquiring essential nutrients, delivering bacterial products, and pathogenicity (Tanaka et al., 2018). ABC transporters can function as either importers or as exporters, which use the binding and hydrolysis of ATP to translocate molecules across the membrane (Rees et al., 2009). The ABC transporters upregulated in response to wastewater effluent are associated with the uptake of peptides involved in the metabolism of amino acids and derivatives. In contrast, the ABC transporters associated with glycerol, tungstate, maltodextrin, and antimicrobial peptides were significantly downregulated after exposure to wastewater effluent.

The TRAP transporters use ion-electrochemical gradients to move substrates across the membrane (Rosa et al., 2018). RNA-seq revealed that TRAP transporters were significantly upregulated in all wastewater effluent conditions but were significantly higher in 100% effluent. Also, the tight adherence (*tad*) genes were significantly upregulated in presence of wastewater effluent: *tadA* (fig|1219061.36.peg.4365), *tadB* (fig|1219061.36.peg.4364), *tadC* (fig|1219061.36.peg.4363), *tadD* (fig|1219061.36.peg.4362), *tadE* (fig|1219061.36.peg.4361), and *tadV* (fig|1219061.36.peg.4371). The *tad* genes are classified as Type II protein secretion systems and encode for the fimbrial low-molecular-weight protein (Flp) pilus assembly machinery, which mediates adherence to surfaces.

Furthermore, multidrug efflux system components were upregulated in wastewater conditions, especially the Acriflavin RND multidrug efflux transporter (fig|1219061.36.peg.1936). Multidrug efflux pumps can transport diverse molecules, including antibiotics, out of the bacterial cell, thus lowering the intracellular concentrations.

### Virulence Factors

**Figure 2:**
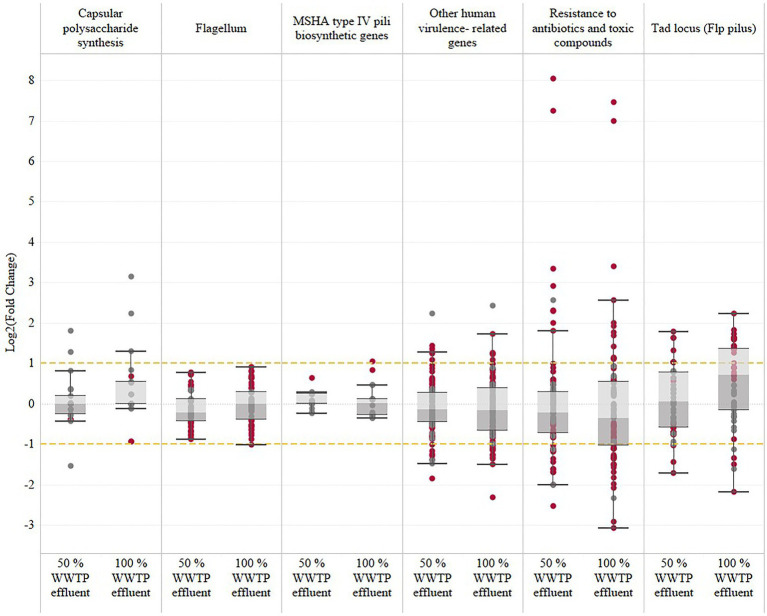
*Vibrio vulnificus* expression of human-virulence-related genes in response to wastewater effluent exposure. The human-virulence genes documented in this study and in previous studies (Williams et al., 2014; Roig et al., 2018) have been identified and categorized into six subcategories based on the most relevant virulence factors: capsular polysaccharide synthesis, flagellum, MSHA type IV pili biosynthetic genes, resistance to antibiotics and toxic compounds, and other human-virulence-related genes. The significantly regulated genes (FDR-adjusted value of *p*≤0.05) are represented with garnet dots, and gray dots represent the non-significant genes.

The expression of human-virulence-related genes previously described by Williams et al. (2014) and Roig et al. (2018) was examined to better understand the potential influence of wastewater effluent on *Vibrio* pathogenicity. Genes encoding flagellum (motility), Flp pilus (*tad* genes), MSHA type IV pili, toxic compounds, and resistance to antibiotics are considered in this analysis (Figure 2). *Vibrio vulnificus* growth is regulated by the cyclic-di-GMP signaling pathway, which allows the bacterium to switch between planktonic and biofilm growth and between constant attachment and dispersion phases (Rinaldo et al., 2018). High cyclic-di-GMP levels serve as a trigger for biofilm formation, leading to the initial step of attachment. Several genes related to adherence (*tad* genes: *tadA*, *tadB*, *tadC*, *tadD*, *tadE*, *tad*F, and *tadG*) and the capsular polysaccharide synthesis (CPS) were upregulated in all wastewater conditions. The *tad* (tight adherence) genes encode a macromolecular transport system required for the biogenesis of Flp pilus. The *tad* genes are essential for biofilm formation, colonization, and pathogenesis of *V. vulnificus*. One gene associated with the MSHA type IV pili, *mshA*, was upregulated only in the 100% wastewater condition.

The NO dioxygenase, HmpA, was the overall highest expressed gene in both wastewater conditions and encodes a flavohemogloblin that is often considered a virulence factor and is induced upon exposure to nitric oxide (Kim et al., 2019).

## Discussion

**Figure 3:**
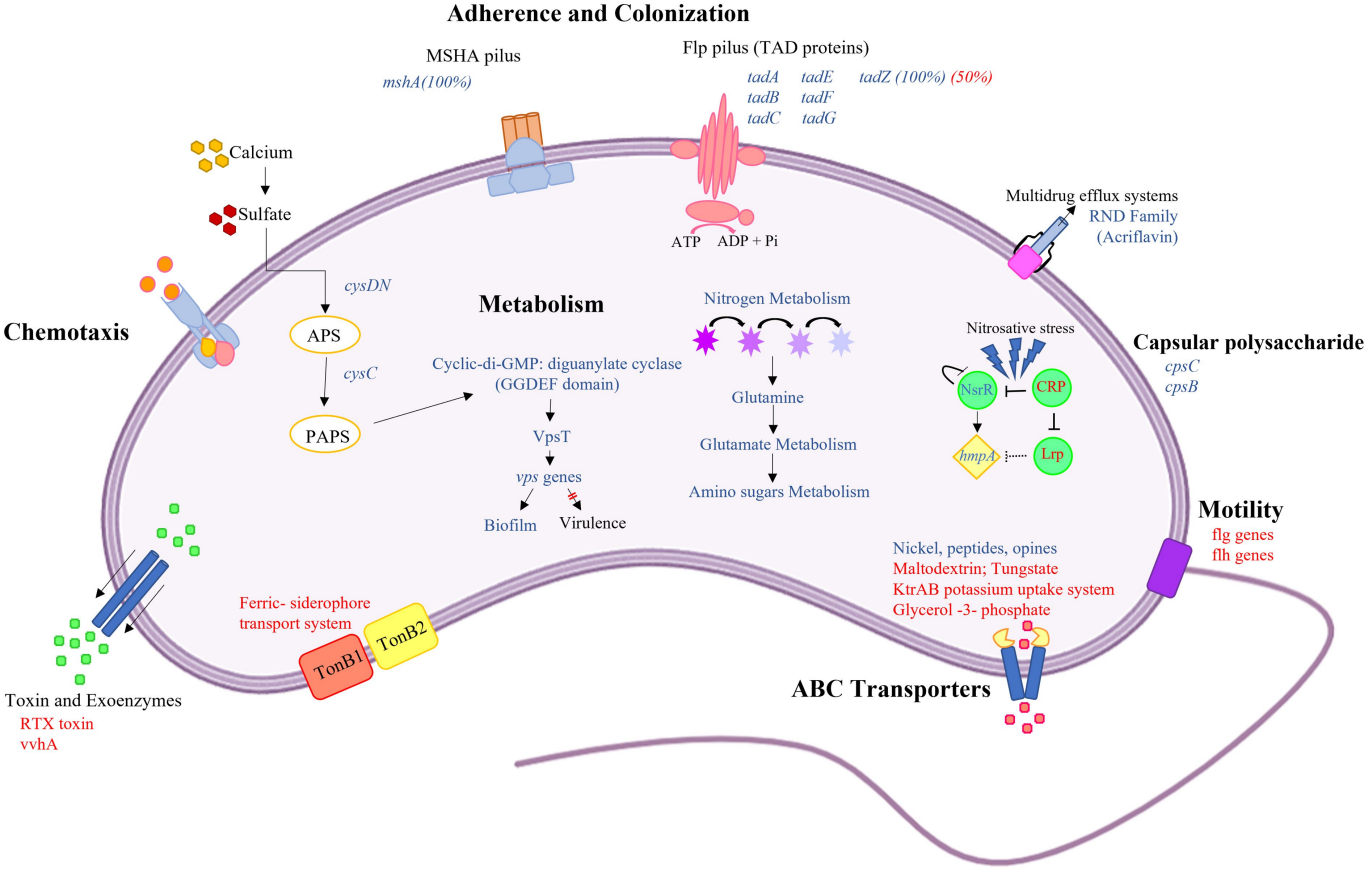
Graphical summary of altered *Vibrio vulnificus* gene expression or pathways with exposure to wastewater effluent. This graphic shows the differentially expressed genes (FDR-adjusted value of *p*≤0.05) in *V. vulnificus* exposed to wastewater effluent compared with the 0% wastewater control. The blue and red text represent upregulated and downregulated genes, respectively. Briefly, *V. vulnificus* exposed to effluent increased transcripts related to attachment and biofilm formation, including Flp pilus (*tad* genes) and MSHA pilus. This transition from planktonic to biofilm formation is driven by the action of diguanylate, which resulted in a high level of intracellular cyclic-di-GMP. This switch in growth state is further supported by the downregulation of flagellar genes (motility) and upregulation the sulfate assimilation genes *cysDN* and *cysC* by calcium as primary environmental signal. In addition, the genes associated with nitrogen metabolism are upregulated suggesting exposure to wastewater effluent alters bacterial metabolism. Increased transcripts for genes involved in substrate binding and transport systems, such as ATP-binding cassette (ABC) transporter and multidrug efflux systems, suggest increased potential for substrate translocation. Lastly, upregulation of the *hmpA* gene suggests that exposure to wastewater effluent acts as a source of nitrosative stress, possibly due to increased nitrogen metabolism.

*Vibrio vulnificus* is a multi-host pathogenic bacterium that can affect humans and marine species, such as fish and crustaceans. In the last decades, *Vibrio* spread and proliferation have been reported in coastal zones and with increased poleward movement (Baker-Austin et al., 2018; Deeb et al., 2018; Froelich and Daines, 2020). These temporal and spatial expansions of *Vibrio* into areas experiencing increased anthropogenic pollution will increase the potential risk of human exposure to *Vibrio* spp. with enhanced virulence profiles. To better understand this emerging public health risk, studies are needed to determine how population-driven increases in pollutants, such as municipal wastewater discharge into coastal zones, may affect bacterial pathogenesis. This study aimed to identify how exposure to wastewater effluent alters *V. vulnificus* gene expression. Figure 3 provides a summary of the main differentially expressed genes identified in wastewater effluent-amended cultures that influence *V. vulnificus* growth, metabolism, and virulence.

The transcriptomic analysis completed in this study showed a shift in the transcript profile suggesting that exposure to wastewater effluent stimulated a transition in *V. vulnificus* to a biofilm mode of growth. The increase in diguanylate cyclase transcripts that was observed in effluent-amended cultures as compared to the no effluent controls suggests increased intracellular levels of cyclic-di-GMP. Cyclic-di-GMP is an intracellular second messenger that has been shown to integrate environmental signals and regulate several cellular processes, enhancing biofilm formation by regulating the production of extracellular surface polysaccharides and repression of motility (Nakhamchik et al., 2008). High intracellular levels of cyclic-di-GMP are associated with biofilm formation, while low levels drive biofilm dispersion (Rinaldo et al., 2018). For instance, in *Vibrio cholerae*, high levels of cyclic-di-GMP have been shown to induce VpsT, a LuxR type regulator that activates the *vps* genes involved in the synthesis of exopolysaccharides used in biofilm development (Wang et al., 2014). Upregulation of *brpT* and *brp* genes (*V. vulnificus* homologs of *vpsT* and *vps*, respectively) was also observed in wastewater effluent-amended *V. vulnificus* cultures, providing evidence that wastewater effluent stimulates a transition to a biofilm mode of growth. The upregulation of this genetic pathway and transition to biofilm growth upon exposure to wastewater effluent is likely due to increased levels of calcium provided by the effluent. Calcium concentrations have been shown to influence biofilm formation by *V. vulnificus* and other non-cholera vibrios (Park et al., 2015; Marsden et al., 2017). It has been proposed that calcium acts as a primary environmental signal by upregulating the sulfate assimilation genes *cysDN* and *cysC*, resulting in the accumulation of 3′-phosphoadenosine 5′-phosphosulfate (PAPS; Chodur et al., 2018). The accumulation of PAPS, in turn, triggers increased intracellular cyclic-di-GMP, resulting in enhanced biofilm formation. The *cysDN* and *cysC* genes were upregulated in the wastewater effluent conditions, suggesting a similar feed-forward loop may be triggering the transition to biofilm growth under effluent exposure. Another factor that regulates biofilm formation and size is the synthesis of capsular polysaccharide (CPS). CPS has been shown to be important in the initial cell adhesion step of biofilm formation as well as the maintenance of mature biofilms (Costerton et al., 1987; Balestrino et al., 2008; Lee et al., 2013; Wang et al., 2015; Çam and Brinkmeyer, 2020). However, another study by Joseph and Wright (2004) suggested that CPS production in *V. vulnificus* inhibits the adherence of planktonic cells, resulting in the inhibition of biofilm formation. The transcriptomic data presented in this work showed upregulation of the CPS genes (*cpsC* and *cpsB*) when *V. vulnificus* was grown in the presence of wastewater effluent. The co-upregulation of these genes with those involved in the synthesis of cyclic-di-GMP, exopolysaccharides, and pili suggests that under the wastewater effluent conditions CPS is aiding in the initial phase of biofilm development. Additionally, the upregulation of the *tad* genes provides further support of effluent-stimulated biofilm formation. These genes encode the machinery needed to assemble the Flp pilus, which is essential for adherence, promoting biofilm establishment and resistance to the complement-mediated bactericidal activity of its host during infection (Duong-Nu et al., 2019). The Flp pilus has also been shown to be involved in host colonization and pathogenesis during *V. vulnificus* infections. Overall, these patterns of gene expression suggest that exposure to wastewater effluent triggers *V. vulnificus* biofilm formation. Similar results have been observed in *V. vulnificus* cultures exposed to estuarine conditions (artificial seawater) wherein genes involved in stress response, chemotaxis, adhesion, and biofilm formation were upregulated (Williams et al., 2014).

In addition to a shift in the mode of growth, the transcript data also suggest changes in *V. vulnificus* metabolism upon exposure to wastewater effluent. The upregulation of nitrate/nitrite reductase and glutamine synthetase genes suggests that the additional nitrogen supplied to the growth media by the wastewater effluent stimulated a shift to a nitrogen-based metabolism. These findings corroborate another study that also observed upregulation of nitrogen cycle genes in *V. vulnificus* exposed to sewage (Zhang et al., 2017). The increased potential for nitrogen metabolism in wastewater effluent-amended cultures also correlated with increased expression of genes involved in *V. vulnificus* nitrosative stress response. Nitrosative response genes not only protect against NO produced during nitrogen metabolism (Vine and Cole, 2011) but are also crucial in overcoming nitrosative stress generated by host immune cells during the infection process (Stern et al., 2012). For instance, the expression of VvHmpA flavohemoglobin, a NO dioxygenase, affects *V. vulnificus* virulence by allowing the bacteria to detoxify the NO produced by the host innate immune system expressed in phagocytes and epithelial cells, thus enhancing bacterial survival and host colonization during infection. In an *in vivo* experiment, mice infected with a VvhmpA mutant had a prolonged survival time, indicating that the deletion of *hmpA* attenuated the virulence of *V. vulnificus* (Kim et al., 2019). Furthermore, another study suggested that NsrR acts as a strong regulator of the *hmpA* gene along with the leucine-responsive protein (Lrp) and the cyclic AMP receptor protein (CRP). The role of these transcriptional regulators in the *hmpA* regulation was evaluated by exposing a *V. vulnificus lrp*-deletion mutant and a *crp*-deletion mutant to elevated NO concentration. The results of these studies suggest that CRP indirectly regulates *hmpA* through repression of *nsrR* and Lrp by enhancing repression activity of NnrR through DNA structure remodeling (Choi et al., 2021). In the wastewater effluent-amended cultures, *lrp* and *crp* genes were downregulated while *nsrR* and *VvhmpA* genes were upregulated, suggesting that effluent may enhance the virulence potential of *V. vulnificus*.

In conclusion, the upregulation of the different metabolic pathways and the transition to *V. vulnificus* biofilm growth upon exposure to wastewater indicated a potential impact on *Vibrio*-human interaction within coastal regions receiving wastewater effluent discharge due to the possible increase in *Vibrio* pathogenicity. This research has shown that the wastewater effluent influenced *Vibrio* growth and metabolism, activating genes, and pathways involved in bacterial survival during early stages of infection in a host, such as biofilm growth, *tad* genes, and nitrosative stress response. The upregulation of these genes due to the interaction with the wastewater effluent and the impact of climate change in the *Vibrio* spread and proliferation may support increased *Vibrio* infections in coastal regions. While this research established a baseline for the effects that wastewater effluent may have on *V. vulnificus* growth and virulence, future experiments should include cultures of different *Vibrio* species grown under simulated climate change conditions and include non-filtered wastewater effluent. By understanding the mechanisms through which climate change and population growth may alter *Vibrio* abundance, distribution, and pathogenicity, better models can be developed to predict future risk of exposure to environmental reservoirs of pathogenic *Vibrio*. It also can provide critical data that inform better policies aimed at mitigating this growing public health risk.

## Data Availability Statement

The datasets presented in this study can be found in online repositories. The names of the repository/repositories and accession number(s) can be found at NCBI [accession: PRJNA747618].

## Author Contributions

KCV and RN conceived and designed the study, analyzed the data, corrected the draft, built the final version of the manuscript, and read and approved the submitted version. KCV performed the lab experiments and wrote the first draft of the manuscript. All authors contributed to the article and approved the submitted version.

## Funding

This work has been funded by the NIEHS Center for Oceans and Human Health and Climate Change Interactions at the University of South Carolina (grant # P01ES028942).

## Conflict of Interest

The authors declare that the research was conducted in the absence of any commercial or financial relationships that could be construed as a potential conflict of interest.

## Publisher’s Note

All claims expressed in this article are solely those of the authors and do not necessarily represent those of their affiliated organizations, or those of the publisher, the editors and the reviewers. Any product that may be evaluated in this article, or claim that may be made by its manufacturer, is not guaranteed or endorsed by the publisher.
