# Supplementary material for: Transcriptomic Analysis Reveals That Municipal Wastewater Effluent Enhances Vibrio vulnificus Growth and Virulence Potential
Source: Front Microbiol. 2021 Oct 25;12:754683. doi: 10.3389/fmicb.2021.754683 (PMC8573347; doi:10.3389/fmicb.2021.754683)
Supplement: Supplementary file 1 [file Data_Sheet_1.docx]

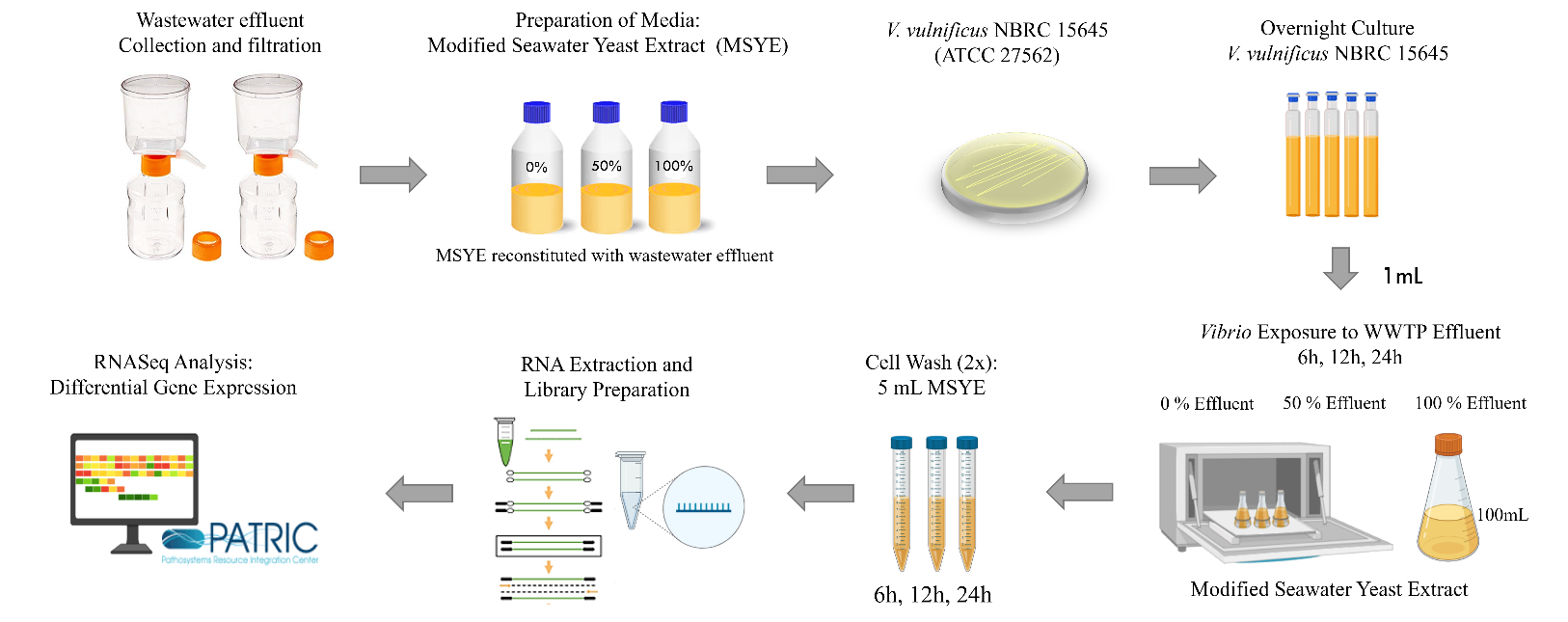


**Figure S1. Graphical methodology of *Vibrio vulnificus* wastewater effluent exposure transcriptomic study.** To assess changes in *V. vulnificu*s gene expression, *V. vulnificus* NBRC 15645 was grown on Modified Seawater Yeast Extract broth containing a range of different effluent concentrations (0%, 50%, and 100%) for 24 hours at 25ºC, and three replicates were performed for each condition. After 6, 12, and 24 hours of incubation, five milliliters of culture were collected and washed twice with MSWYE to remove any residue of wastewater that could interface with the subsequent process. RNA was extracted using Allprep Bacterial DNA/RNA/Protein kit (Qiagen) follow the manufacture's instructions. RNA quality and quantity were assessed, and ribosomal RNA was removed from the total RNA to proceed with the library preparation. DNA libraries complementary to mRNA were prepared using NEBNext Ultra II Directional RNA Library Prep Kit according to the manufacturer's instructions for intact RNA. The cDNA libraries were sequenced on an Illumina HiSeq DNA sequencer with 2 x 250bp paired-end reads. Following sequencing, raw reads were trimmed, and the cleaned RNA-seq sequences were aligned with a reference, annotated, and analyzed to find differentially expressed genes using the Tuxedo strategy in the Pathosystems Resource Integration Center (PATRIC) analysis tool.

**Figure S2**. **Bacterial growth of *Vibrio vulnificus* NBRC 15645 (ATCC 27562) in Modified Seawater Yeast Extract broth containing 0%, 50%, and 100% effluent.** The exposure of the *V. vulnificus* NBRC 15645 (ATCC 27562) into 0% (blue), 50% (orange), and 100% (gray) effluent show enhanced growth with wastewater presence. The solid lines represent a pilot study where *V. vulnificus* was grown in the experimental conditions in a 96 well plate per 24 hours. The dotted lines represent the bacterial growth during the transcriptomic exposure study, where the optical density was measured at 0h, 6h, 12h, and 24h. A similar bacterial growth pattern was observed in both experiments.


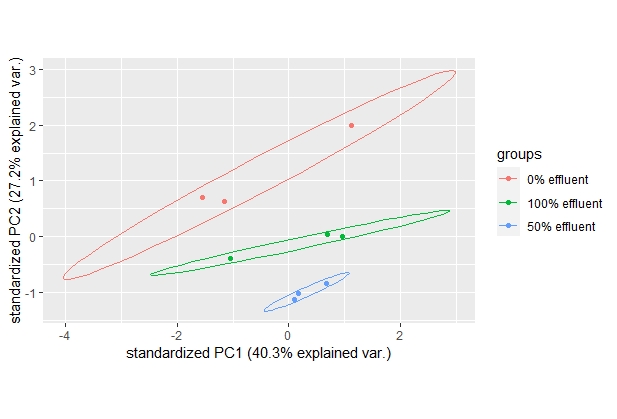


**Figure S3. Principal component analysis (PCA) plot of the global gene expression profile of *Vibrio vulnificus* NBRC 15645 (ATCC 27562) in Modified Seawater Yeast Extract broth containing 0%, 50%, and 100% effluent**.. The ellipses represent at 68% confidence interval.

**
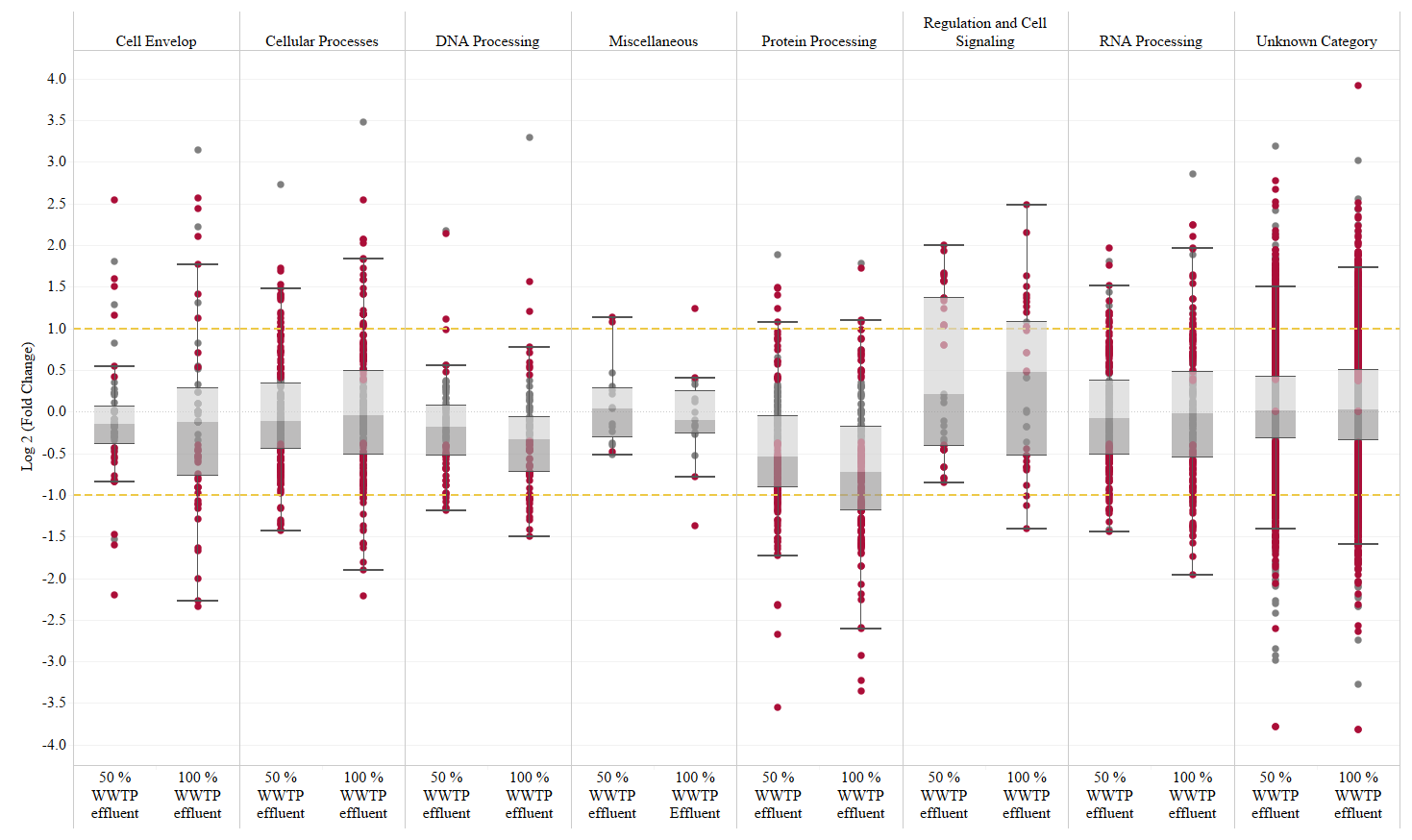
**

**Figure S4. Summary of *V. vulnificus* transcriptomic response to wastewater effluent across additional categories.** This table shows the overall log_2_ fold change at 50% and 100% wastewater effluent relative to the 0% wastewater effluent control. The significantly regulated genes (FDR-adjusted p-value ≤ 0.05) are represented with garnet dots, and gray dots represent the non-significant genes.

**
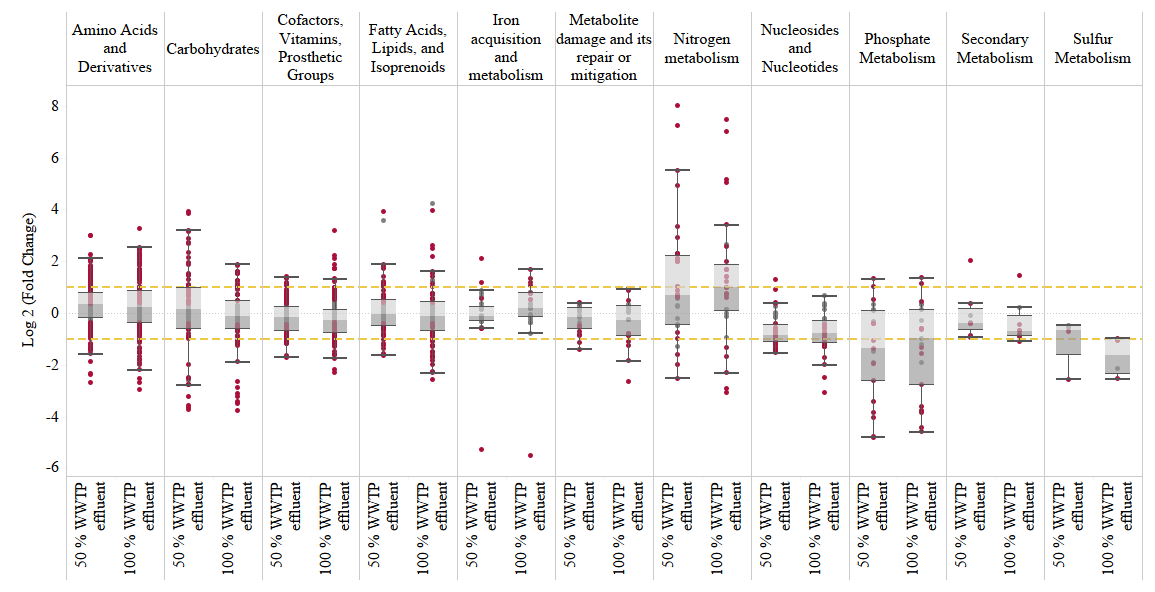
Figure S5**. Summary of fold change in metabolism-related genes by subcategories for *V. vulnificus* exposed to wastewater effluent.

**
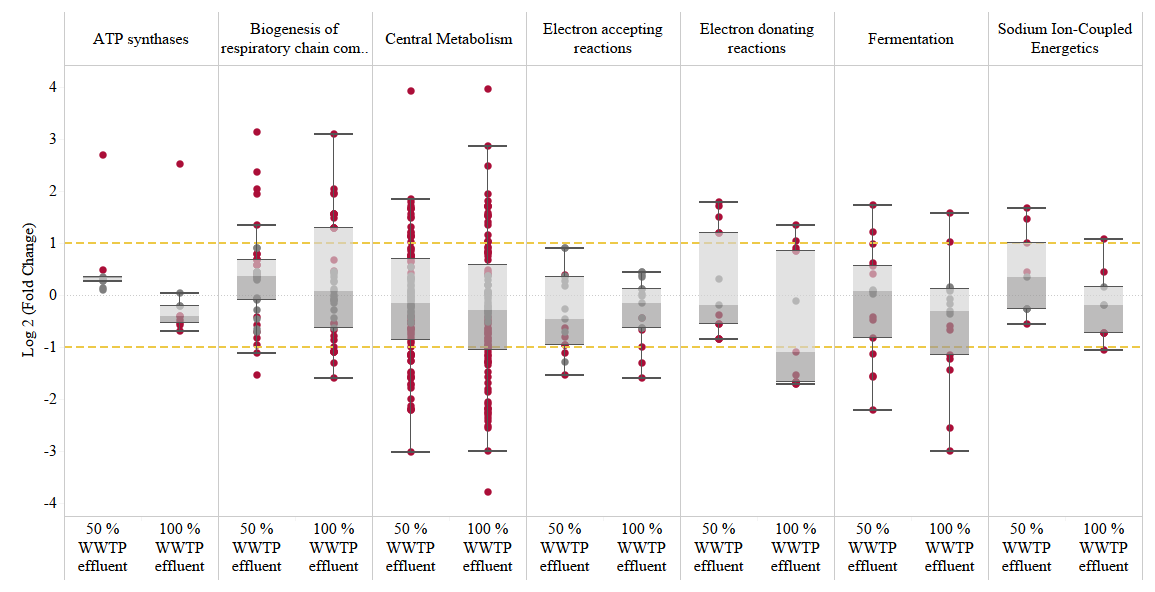
****Figure S6**. Summary of fold change in energy-related genes by subcategories for *V. vulnificus* exposed to wastewater effluent.

**
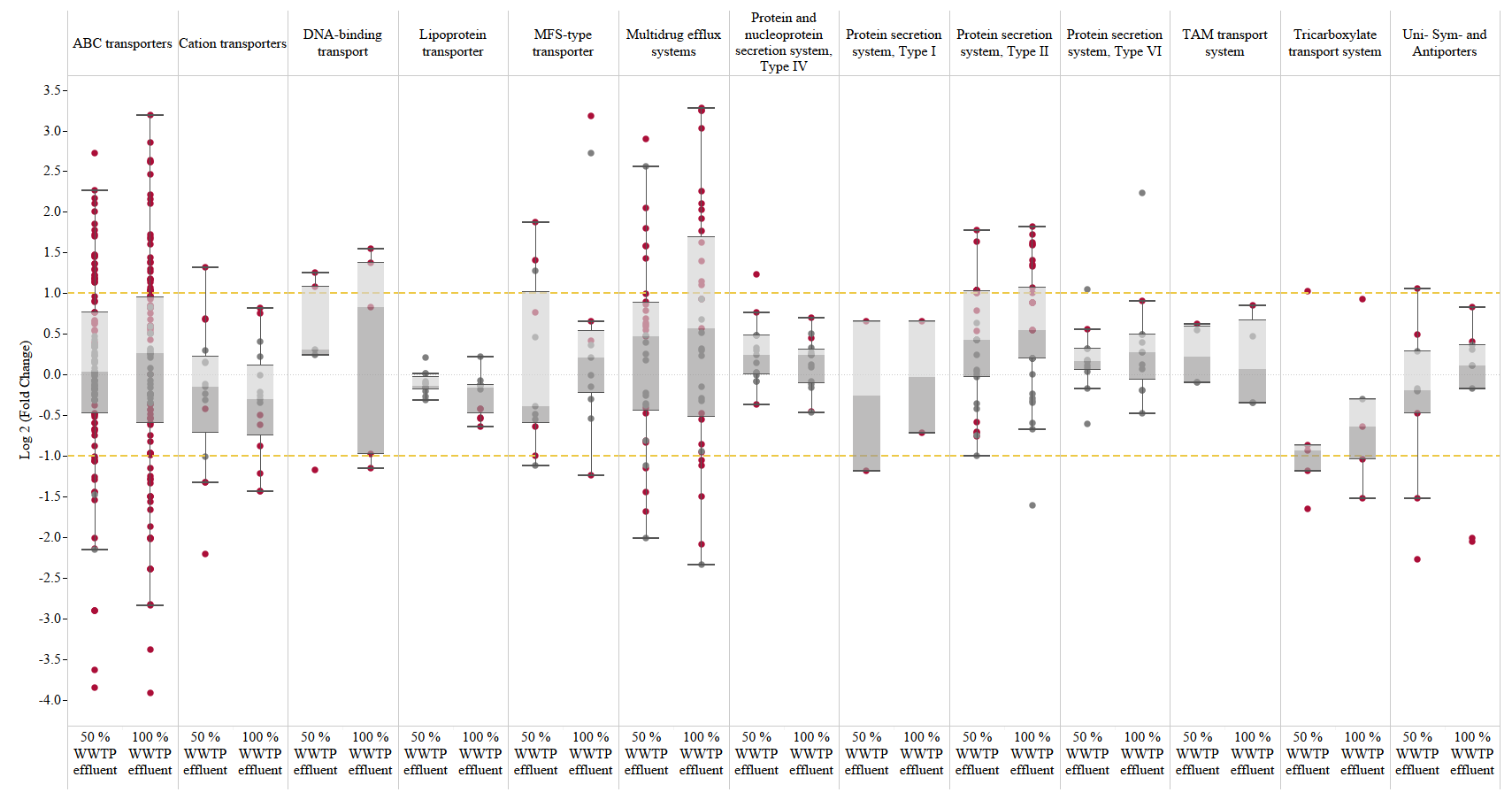
Figure S7**. Summary of fold change in membrane transport-related genes by subcategories for *V. vulnificus* exposed to wastewater effluent
